# Reply to Venturino et al. Comment on “Hladky-Krage, B.; Hoffman, C.L. Expectations versus Reality of Designer Dog Ownership in the United States. *Animals* 2022, *12*, 3247”

**DOI:** 10.3390/ani15111519

**Published:** 2025-05-23

**Authors:** Bridget Hladky-Krage, Christy L. Hoffman

**Affiliations:** 1 Department of Animal Behavior, Ecology, and Conservation, Canisius University, Buffalo, NY 14208, USA; 2 Independent Researcher, Buffalo, NY 14208, USA; christyhoffman@gmail.com

We appreciate Venturino, Weaver, Kraus, and Hekman’s comment on our paper “Expectations versus Reality of Designer Dog Ownership in the United States” [1,2]. We have broken our response into the sections they highlighted.

## 1. Better Health in Crossbred Dogs?

The authors requested the results regarding whether veterinary costs met expectations. We have included those results below (Table 1). This table displays the percentage of dog owners who reported exercise requirements, veterinary costs, and overall behaviors as “worse than expected”. There were no significant differences between the three groups regarding veterinary costs or exercise requirements; however, fewer doodle owners than owners of purebreds and mixed breeds reported their dogs’ overall behaviors as being worse than expected (χ^2^ = 24.56, df = 2, *p* < 0.001; post hoc comparisons: doodle vs. purebred: *p* < 0.05; doodle vs. mixed breed: *p* < 0.001; mixed breed vs. purebred: *p* > 0.05).

Venturino et al. describe conclusions from some prior studies that suggest mixed-breed dogs tend to be healthier than purebred dogs [1]. As Venturino et al. point out [1], Bellumori and colleagues’ study found 10 diseases with a higher prevalence in purebred dogs, 1 disease which had a higher prevalence in mixed-breed dogs, and 13 diseases for which there was no difference [3]. Bellumori and colleagues also explain that mixed-breed dogs can inherit the same diseases as purebred dogs, specifically stating that “every disorder was seen in the mixed-breed population. Thus, on the basis of the data and analyses, the proportion of mixed-breed and purebred dogs affected by genetic disorders may be equal or differ, depending on the specific disorder” [3] (p. 1553). This quote highlights why we were intentional in stating that “being crossbred does not *inherently* make a dog healthier” [2] (p. 2). Bellumori et al. also explain that “the prevalence of disorders among purebred and mixed-breed dogs in the present study depended on the condition” [3] (p. 1554). This same idea is echoed in the resource that Venturino and colleagues suggested [1], in which Michell found that mixed-breed dogs lived longer than average; nevertheless, they also reported that several breeds lived longer than mixed breeds [4]. Other studies have found that crossbred dogs lived on average 1.2 years longer than purebreds [5,6], suggesting that crossbreeding dogs can produce improvements in health and fitness [6]. This idea, however, is complicated. Farrell et al. [7] explain that though outcrossing can increase heterozygosity, it is essential to avoid lineages that have shared genetic diseases. The authors specifically mention that Labradors’ and Standard Poodles’ shared afflictions include hip dysplasia and eye and joint diseases. Additionally, they caution that health benefits seen in the first generation (F1) of crossed breeds can deteriorate in subsequent generations if breeders do not maximize genetic diversity and avoid inherited conditions in future crosses [7]. Finally, a study published subsequent to our 2022 paper has concluded that Cockapoo, Labradoodle, and Cavapoo designer crossbreeds are neither healthier nor less healthy than their purebred progenitors [8].

## 2. A Family-Friendly Temperament?

Venturino et al. reference our finding that “actual owner satisfaction was significantly higher with doodles than with the other groups tested, including owners with children in the home, which were twice as frequent as both purebred and mixed breed dogs” [1]. They inquired how satisfaction scores of participants with children differed across the three groups of dog owners. To address this question, we performed a Kruskal–Wallace test, which indicated there were no significant differences in the behavior scores across the three groups of dog owners (χ^2^ = 0.224, df = 2, *p* = 0.894). Doodle owners, however, still reported higher satisfaction scores compared to mixed-breed and purebred owners (χ^2^ = 12.35, df = 2, *p* = 0.002). Additionally, Venturino et al. [1] requested the results from Section I of the survey, which examined expectations being met regarding exercise levels and overall behavior. These results are also included in Table 1. We also ran a Kruskal–Wallace test comparing barking behavior reported by owners of the different breed groups and found no significant difference across groups (χ^2^ = 3.38, df = 2, *p* = 0.19). Although doodle owners more commonly reported excessive barking in response to the survey’s open-ended questions, they did not score their dogs differently on barking compared to mixed-breed or purebred owners.

Regarding the qualitative analysis, the authors also state that “The paper only included discussion of a qualitative analysis for the responses provided by doodle owners. We suggest adding a comparison to the responses from purebred and mixed-breed owners, including numbers of answers from each group” [1]. Readers will see that we did include this information on page 9 of the original paper. On this page we state, “Of the doodle owners who answered the free-response question, 87 (18.2%) doodle owners lamented about the cost and frequency of grooming, and 32 (6.7%) complained specifically about how much daily brushing was required, stating that they expected their dog to have a low-maintenance coat and instead ended up with the opposite. Comparatively, 20 (3.1%) owners of mixed-breed dogs complained of their dogs’ grooming needs, and 9 (1.4%) lamented their dogs’ general maintenance. Of the purebred owners who answered the question, 25 (5.2%) complained of their dogs’ grooming needs, and 21 (4.4%) complained of their dogs’ general maintenance” [2]. In the next paragraph, we continue with, “Complaints about grooming were second only to negative statements about barking. Of the 479 doodle owners who answered the free-response question about how their dog did not meet their expectations, 122 (25.5%) stated that their dog barked more than expected. In comparison, out of the 637 mixed-breed owners and 479 purebred owners who answered the question, 118 mixed-breed owners (18.5%) and 81 purebred owners (16.9%) listed barking as an expectation not met” [2] (p. 9).

## 3. Doodle Coat Maintenance and Allergenicity

Though we did include background information regarding the perceived allergenicity of doodles in our introduction, investigating allergenicity was outside the scope of this project. Further research would be needed to explore how marketing doodles as hypoallergenic affects motivations to purchase a doodle and owner satisfaction with doodles post-purchase.

Venturino et al. [1] are correct that we inadvertently omitted the question about maintenance levels from the Supplementary Materials. The question asked respondents to indicate whether “maintenance levels (physical maintenance, e.g., grooming)” were “better than expected”, “met expectations”, or “worse than expected”.

## 4. Pre-Purchase Motivations of Doodle Owners

Venturino and colleagues correctly state that appearance was only part of what motivated participants to purchase a doodle, as participants were also influenced by the perception that doodles are good with kids (53.7%), good companion breeds (83.2%), a generally healthy breed (51.7%), and suited to their lifestyle (69.8%) [1,2]. However, it also remains true that doodle owners prioritized appearance (49.9%) significantly more than owners of purebreds (36.7%) and mixed breeds (30.0%).

Although we do see the authors’ point that appearance was one of several factors influencing doodle owners’ decisions, some of our other findings do suggest that health and behavior were not fully considered. For instance, fewer owners of doodles than owners of purebred dogs met at least one of their dog’s parents before acquiring the dog (57.1% of doodle owners compared to 67.1% of purebred owners). Meeting a puppy’s parents can give prospective dog owners crucial information, such as the parents’ temperaments, interactions between the mother and puppies, the general conditions of the dogs, and signs of distress or poor maternal care [9]. These observations are also important when considering heritable behavioral traits such as aggression and fearfulness [10,11,12]. Additionally, breeder concealment of the mother can indicate poor breeding conditions [9]. Thus, our finding that fewer doodle owners met their dog’s parents than purebred owners provides evidence that doodle owners are not as commonly taking steps to verify the health and behavior of the dogs they purchase.

## 5. Non-Doodle Designer Dogs

Non-doodle designer dogs were an extremely small subset of our study sample (only 56 respondents out of 2191 total, or 2.6%). Many of these 56 responses described types of dogs that were unique, the only of their kind in the dataset, and much less common in general than doodles. This made it difficult to compare those dogs with our large dataset of doodles. Examples of these uncommon hybrids include a mastiff/Labrador mix, a St. Bernard/Great Dane mix, a Golden Retriever/Australian Shepherd mix, a Cocker Spaniel/Cavalier King Charles Spaniel mix, and a Pomeranian/husky/heeler mix. Thus, we combined this group of dogs with the mixed-breed group.

## 6. Literature and Language Use

As our study was mixed-methods and included a large amount of qualitative data, including a *Newsweek* article, along with small-scale interview studies, for background information is entirely appropriate. The inclusion of the article from *The Guardian* as a source was also intentional and is essential to the background information shared in our paper [13]. This article included an interview of Wally Conron, the individual credited with “creating” the Labradoodle and starting the trend of crossing poodles with many other dog breeds. The quotes we included convey Conron’s feelings regarding the trend of breeding designer dogs [12]. Finally, our use of the word “fad” is simply descriptive rather than an indication of bias. The term reflects the extreme, sudden popularity of doodles in recent years. The use of this term in relation to the popularity of dog breeds is not new and can be found in other papers such as Herzog, 2006 [14].

## 7. Conclusions

We appreciate the amended set of conclusions proposed by Venturino and colleagues [1]. However, these ideas are indeed reflected in our original publication. Just as Venturino et al. [1] suggest, we did state that doodle owners were highly satisfied with their dogs. We also highlight that the main difference between doodle owners and the other groups was that they were more likely to report that the maintenance requirements of their dogs were worse than expected. Additionally, we concluded that breeders could do more to properly educate potential owners on maintenance and grooming [2] (pp. 10–11). We do agree that further research is needed, such as investigation into the allergenicity of different coat types. Questions such as this were beyond the scope of our research, but we look forward to future research that others perform.

## Figures and Tables

**Table 1:** The percentages of doodle owners, purebred owners, and mixed-breed owners that reported their dogs’ veterinary costs, exercise requirements, and overall behaviors as “worse than expected”.

Variable	Doodles	Purebreds	Mixed Breeds
Veterinary Costs	17.47%	20.15%	21.38%
Exercise Requirements	7.57%	7.85%	8.25%
Overall Behaviors	13.10%	18.92%	23.12%

## Data Availability

The data presented in this study are available upon request from the authors.
